# Seasonal Dynamics of *Microcystis* spp. and Their Toxigenicity as Assessed by qPCR in a Temperate Reservoir

**DOI:** 10.3390/md9101715

**Published:** 2011-09-29

**Authors:** António Martins, Cristiana Moreira, Micaela Vale, Marisa Freitas, Ana Regueiras, Agostinho Antunes, Vitor Vasconcelos

**Affiliations:** 1 CIIMAR/CIMAR, Marine and Environmental Research Centre, Porto University, Rua dos Bragas, 289, Porto 4050-123, Portugal; E-Mails: amartinstz@net.sapo.pt (A.M.); cmoreira@ciimar.up.pt (C.M.); micaela.vale@ciimar.up.pt (M.V.); maf@estsp.ipp.pt (M.F.); anaregueiras@gmail.com (A.R.); aantunes777@gmail.com (A.A.); 2 Department of Biology, Faculty of Sciences, Porto University, Rua do Campo Alegre, Porto 4069-007, Portugal; 3 CISA/Research Centre in Environment and Health, School Health Technology of Oporto, Polytechnic Institute of Porto, Rua de Valente Perfeito, 322, Gaia 440-330, Portugal

**Keywords:** microcystins, *Microcystis*, qPCR, water quality monitoring, water management

## Abstract

Blooms of toxic cyanobacteria are becoming increasingly frequent, mainly due to water quality degradation. This work applied qPCR as a tool for early warning of microcystin(MC)-producer cyanobacteria and risk assessment of water supplies. Specific marker genes for cyanobacteria, *Microcystis* and MC-producing *Microcystis*, were quantified to determine the genotypic composition of the natural *Microcystis* population. Correlations between limnological parameters, pH, water temperature, dissolved oxygen and conductivity and MC concentrations as well as *Microcystis* abundance were assessed. A negative significant correlation was observed between toxic (with *mcy* genes) to non-toxic (without *mcy* genes) genotypes ratio and the overall *Microcystis* density. The highest proportions of toxic *Microcystis* genotypes were found 4–6 weeks before and 8–10 weeks after the peak of the bloom, with the lowest being observed at its peak. These results suggest positive selection of non-toxic genotypes under favorable environmental growth conditions. Significant positive correlations could be found between quantity of toxic genotypes and MC concentration, suggesting that the method applied can be useful to predict potential MC toxicity risk. No significant correlation was found between the limnological parameters measured and MC concentrations or toxic genotypes proportions indicating that other abiotic and biotic factors should be governing MC production and toxic genotypes dynamics. The qPCR method here applied is useful to rapidly estimate the potential toxicity of environmental samples and so, it may contribute to the more efficient management of water use in eutrophic systems.

## 1. Introduction

Blooms of toxic cyanobacteria are a growing problem in freshwater ecosystems due to water quality degradation caused by eutrophication [1,2]. Toxic blooms pose significant health risks to water users and may prevent its recreational use [3]. Previous research has shown that more than 50% of freshwater blooms produce toxins, with hepatotoxic blooms being more common than neurotoxic ones [4]. Microcystins (MCs) are the most prevalent cyanobacterial hepatotoxins in freshwaters and are produced mainly by strains of *Anabaena*, *Microcystis* and *Planktothrix* [4–6]. In Portugal, the occurrence of cyanotoxins is well documented, with MCs being the most prevalent [7,8], and MC-LR, MC-RR, and MC-YR as the dominant variants. *Microcystis aeruginosa*, *M. wesenbergii*, *Anabaena flos-aquae*, *A. scheremetievi* and *Aphanizomenon flos-aquae*, are the dominant toxic cyanobacteria species in most of the water bodies [7–9].

MCs are a chemically diverse group of cyanotoxins and their toxicity is due to inhibition of serine-threonine protein phosphatases 1 and 2A [10], causing both acute and chronic effects in mammals [3,4]. MC acute intoxications lead to hepatocyte necrosis and liver hemorrhage, with severe cases resulting in death [11]. Some evidence of tumor-promoting activity of MC due to long term exposure was also reported [12–14]. MCs are produced non-ribosomally via an integrated multifunctional enzyme complex, consisting of both peptide synthetase and polyketide synthase modules coded by the MC synthetase (*mcy)* gene cluster [15–18].

The most common methods used for monitoring MC are high-performance liquid chromatography with photodiode array detector and enzyme-linked immunosorbent assays (ELISA) [19]. More recently, other high throughput analysis methods such as protein phosphatase inhibition assay (PPIA) and liquid chromatography–mass spectrometry (LC/MS) have become available for MC monitoring [20]. Microscopy is still a valuable first-line monitoring strategy; however, it does not differentiate toxic from nontoxic cyanobacteria, is subject to considerable counting error, and may result in misidentification when there are limited morphological differences [4]. Cyanobacteria known as potential MC-producers comprise both toxic (with the *mcy* genes) and nontoxic strains (without the *mcy* genes). The presence or absence of the *mcy* gene cluster has been used to distinguish the two types of strains [21,22]. In the recent years, quantitative molecular methods have been developed to study the succession of MC-producing genera and monitor toxic blooms formation. Detection and quantification by quantitative real-time PCR (qPCR) of MC-producing cyanobacteria is sensitive and rapid. Furthermore, quantification of MC-producing *Microcystis* genotypes has been proposed as a surrogate for MC-producing cells in natural populations [23–27]. qPCR quantification of specific toxin-producer cyanobacteria can also be a valuable tool in developing genus-targeted restoration strategies [28].

MC concentration in water is mostly dependent on the density of the hepatotoxic cells [4]. Some strains may produce higher concentrations of MCs than others under the same laboratory conditions, while others can be more or less toxic depending on cultivating conditions [29]. In addition, the succession of cyanobacteria species and biomass is influenced by seasonal changes of several factors including nutrients, grazing, light and temperature, which affect the concentration of MC in the field [4,30–32]. The effects of environmental factors on the abundance of MC-producing and non-toxic *Microcystis* genotypes have, however, been studied on a limited scale [24,33–36].

In this work, qPCR was used to evaluate the seasonal dynamics of cyanobacteria populations, particularly *Microcystis* populations, in an aquatic ecosystem from North Portugal, known for the periodic dominance of toxin-producing cyanobacteria, including *Microcystis aeruginosa* [37]. We sampled in a single site where a water treatment plant is located and the results are valid only for this location. We applied a tool that may enable the early warning for MC-producing cyanobacteria presence and thus of potential toxic risk. This methodology will be valuable for water treatment plants management providing information on emerging toxic blooms so that adequate water treatments can be prepared ahead of time. qPCR was applied to quantify specific marker genes for cyanobacteria, *Microcystis* and MC-producing *Microcystis*, and to quantitatively determine the genotypic composition of the natural *Microcystis* population.

## 2. Results and Discussion

pH values ranged from 6.1 (October 1) to 8.8 (July 1), DO from 4.0 mg/L (October 28) to 10.5 mg/L (July 30), water temperature from 20.5 °C (October 28) to 27.0 °C (August 12) and conductivity from 60 μS/Cm (July 1) to 155 μS/Cm (October 28) (Table 1). pH and temperature followed approximately the same pattern both presenting a peak at August 12, and then declining in the following sampling dates. Conductivity presented approximately the same values until the end of August, increasing sharply in the last two sampling dates. This sharp increase refer to the two last samplings that were performed monthly, instead of the bimonthly periodicity of the rest, however, the values were always low. Dissolved oxygen values did not have a clear pattern throughout the sampling period.

### 2.1. Quantitative Analysis of *Microcystis* spp. Community Composition by qPCR

Standard curves were established by conducting serial dilutions (1.2–1.2 × 10^6^), of cyanobacterial 16S rRNA, *Microcystis* 16S rRNA, *mcyA* and *mcyB* gene cell equivalents/mL of genomic DNA extracted from the *M. aeruginosa* M6 culture. For cyanobacterial 16S, *Microcystis* 16S, *mcyA* and *mcyB*, highly significant linear curves between the amount of starting DNA (in cell equivalent numbers) and the threshold cycle values (Ct) were obtained. The efficiencies and curve parameters obtained for the different standard curves are summarized in Table 2.

**Figure 1:**
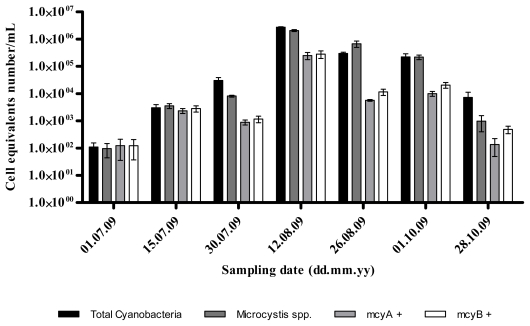
Variations in the numbers of Cyanobacteria 16S rRNA, *Microcystis* 16S rRNA, *mcyA* and *mcyB* genes, expressed as cell equivalents during the sampling period.

The melting temperature of the different qPCR products of the samples were similar to those obtained with the standard strain (Table 2), thus demonstrating the reliability of the real-time PCR amplification. The variation of the number of both cyanobacteria and *Microcystis* spp. followed the same pattern throughout the sampling period progressively increasing after the first sampling date (9.5 ± 5.2 × 10^1^ and 1.1 ± 0.5 × 10^2^ cell equivalents/mL, respectively), reaching the maximum values at August 12 (2.7 ± 0.1 × 10^6^ and 2.1 ± 0.2 × 10^6^ cell equivalents/mL, respectively), and then progressively declining until the last sampling date (7.2 ± 4.0 × 10^3^ and 9.7 ± 5.8 × 10^2^ cell equivalents/mL, respectively) (Figure 1). Both the number of potentially MC-producing cyanobacteria and MC-producing *Microcystis* varied in a similar way throughout the entire sampling period, presenting its maximum value at August 12 (2.5 ± 0.7 × 10^5^ and 2.8 ± 0.9 × 10^5^ cell equivalents/mL, respectively), and the minimum at July 1 (1.2 ± 0.8 × 10^2^ and 1.2 ± 0.8 × 10^2^ cell equivalents/mL, respectively).

**Figure 2:**
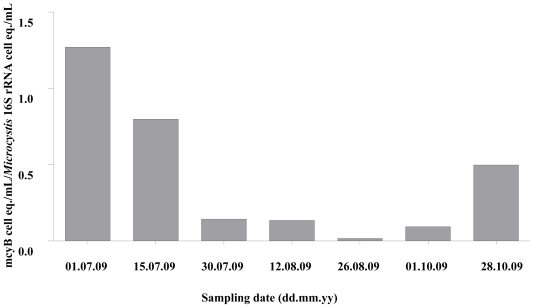
Ratios obtained between number of *mcyB* and *Microcystis* 16S rRNA genes, expressed in cell equivalents, for each sampling date, along the sampling period.

The data obtained by qPCR, were also used to evaluate the dynamics of the proportion of toxic genotypes inside the *Microcystis* spp. population (expressed by the ratio between the number of *mcyB* genes in cell equivalents/mL and the number of *Microcystis* 16S rRNA genes in cell equivalents/mL) throughout the sampling period (Figure 2).

Within the *Microcystis* spp. population, the percentage of potentially MC-producing genotypes varied from around 2% to higher than 100% of the cells. The percentage of toxic genotype progressively and markedly decreased from the first sampling date, at which all the cells of *Microcystis* seem to present the *mcyB* gene, and reaching a minimum of around 2% at August 26. Then, the proportion of toxic genotype increased again, constituting around 50% of the entire population in the last sampling date. This dynamics matched the one observed for the *mcyA* cell equivalents number (data not shown).

**Figure 3:**
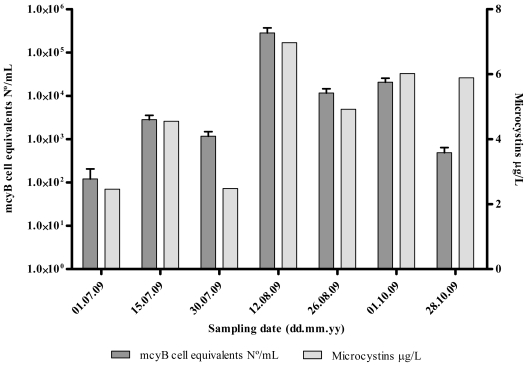
Comparison between the number of *mcyB* genes, expressed in cell equivalents/mL, and MCs concentration for each sampling date along the sampling period.

**Figure 4:**
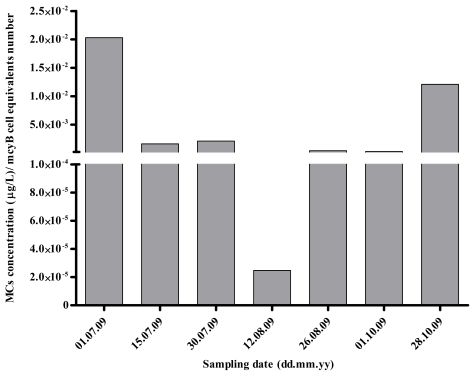

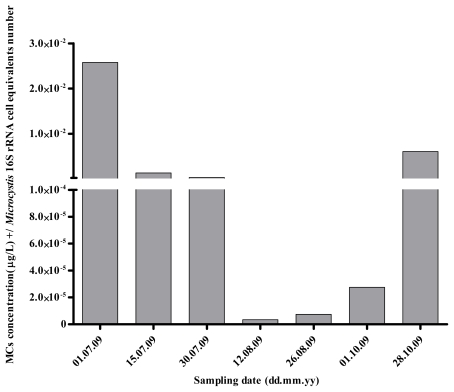
Ratios obtained between MCs concentration values and number of *mcyB* and *Microcystis* 16S rRNA genes, expressed in cell equivalents number/mL, for each sampling date, along the sampling period.

MC quantified by ELISA varied between 2.46 (July 1) and 6.97 μg MC-LR eq/L (August 12). MC values followed approximately the same pattern of the variation as toxic *Microcystis* genotypes (*mcyB*) densities, except in the last sampling date (Figure 3). The highest value of MC was determined for the same date as the highest number of *mcyB* cell equivalents (August 12). Curiously, at this date, we can also observe the lowest MC quota per toxic genotype (*mcyB*) (Figure 4). The MC concentrations were always above the WHO guideline values for drinking water (1 μg MC-LR/L) for all the sampling dates.

MC concentration had a significant positive correlation (*r* = 0.79; *P* < 0.05) with the abundance of *Microcystis mcyB* and of cyanobacterial *mcyA* cell equivalents (Table 3).

Furthermore, strong positive correlations were found between the abundance of *Microcystis mcyB* cell equivalents and the abundance of cyanobacterial *mcyA* cell equivalents (*r* = 1.00; *P* < 0.001), and between the abundances of *Microcystis* 16S rRNA cell equivalents and cyanobacterial 16S rRNA cell equivalents (*r* = 0.96; *P* < 0.05). The proportion of toxic genotypes in the *Microcystis* population correlates negatively, but not significantly, with MC concentration (*r* = −0.61). Moreover, the proportion of toxic genotypes showed a negative significant correlation with both *Microcystis* and cyanobacteria abundances (*r* = −0.89 and −0.86, respectively; *P* < 0.05). Concerning the influence of limnological parameters on the overall MC level, no significant correlations between MC concentrations and pH, DO, water temperature or conductivity were detected (Table 3).

### 2.2. Discussion

The qPCR technique is currently the only quantitative tool available to determine the proportion of potential toxin-producing genotypes in environmental water samples. Although some previous reports have showed a close relationship between the occurrence of certain toxic genotypes (containing toxin genes) and MC net production [38], and that it is possible to infer MC concentrations from *Microcystis* cell numbers [39], correlations between MC levels and toxic genotypes numbers are often difficult to obtain [26,40]. The work presented here aimed to help clarify these contradictory observations. In this work, the proportion of toxic *Microcystis* genotypes varied between approximately 2% and around 100% of the entire *Microcystis* population. These values are above others previously reported between toxic and non-toxic *Microcystis* strains, such as 10% in Lake Kasumigaura (Japan) [41], 34% in Lake Kasumigaura [42], 16% in Lake Grand-Lieu (France) [43], 45% in Lake Wannsee and Lake Pehlitzsee (Germany) [44], ranging from 1 to 38% [39]. More recently, proportions of *mcyB* genotypes ranging from 6% to 93% of the *M. aeruginosa* population were reported in Grangent reservoir, France, with the highest proportions appearing at the beginning and after the decline of the bloom [45]. Our results also revealed an inverse relationship between density and frequency of toxic genotype and the highest *Microcystis* cell densities, with the former being detected 4–6 weeks before and 8–10 weeks after the latter (Figures 1 and 2). Indeed, significant negative correlations were obtained between *mcyB* genotype proportions and cyanobacteria and *Microcystis* cell densities (Table 4). Nevertheless, the highest toxic genotypes numbers and MC concentration values were, in fact, found when the highest *Microcystis* cell densities were observed. *McyB* is specific to *Microcystis* producing MC and *mcyA* is present in all cyanobacteria genera that produce MC. By using these two markers it can be determined whether the MC is mainly produced by *Microcystis*, which was the case, or whether other cyanobacteria are responsible for the toxin production.

Detection of MC-producing genotypes during non-bloom season (winter), has also been previously reported [26], however, no MC production could be detected. This raised the possibility that toxic cyanobacteria do not produce MC during times of low growth or low metabolism (during the winter), although mcy genotypes may be present [26,39]. In the present work, MCs were always detected, with the highest MC cell quotas being observed simultaneously with the lowest *Microcystis* numbers and vice versa (Figures 2 and 4). This may suggest that MC production may play an important role when conditions do not favor *Microcystis* growth. Previous data also showed a negative relationship between cyanobacterial biomass and average MC cell content during the development of cyanobacterial blooms [45].

On the other hand, the observed decrease in toxic genotypes proportion as the bloom developed suggests that favorable environmental conditions for *Microcystis* growth lead to the selection of non-toxic genotypes. In fact, it was previously shown that, under environmental conditions favorable for cyanobacterial growth, the fitness of non-MC-producing strains is greater than that of MC-producing ones [34,47]. Similarly, a negative correlation between cell abundance and the proportion of potentially MC-producing genotypes was observed during a *P. agardhii* bloom [48].

Considering the influence of environmental factors on MC levels and on *Microcystis* population dynamics, in the present work, no significant correlations were found between MC concentration and any of the limnological parameters measured. The regulation of the internal dynamics of MC-producers populations is predictable to be affected by a large array of environment conditions [49]. For instance, the influence of nitrate concentrations on the relative dynamics of *mcy* genotypes was shown within the *M. aeruginosa* communities of Lake Mikata [24]. Furthermore, it was already suggested that grazers play an important role on this dynamics. Due to selective grazing, depending on whether the cell is producing toxin or not, grazers may affect the total biomass of cyanobacteria and *Microcystis*, as well as the relative abundance of *Microcystis* genotypes [26,32]. Other authors suggested that cyanophages may be involved in the interaction between microcystin-producing and non-microcystin-producing *M. aeruginosa* populations [49].

The results now obtained confirm that one of the most known problems of qPCR methodologies is the difficulty in converting the gene quantities to quantities of cells that carry these target genes. This can distort the enumeration of cyanobacterial cells in natural samples by qPCR. In fact, as described previously in other studies [26], some samples showed an abundance of potentially toxic *Microcystis* higher than the total *Microcystis* abundance and in other cases, the abundance of *Microcystis* cells appeared >100% of the total abundance of cyanobacteria. These results may be due to the use of the 16S rRNA gene as a target for quantification of both cyanobacteria and *Microcystis* due to the variable copy number of 16S rDNA operons or their sequence heterogeneity in cyanobacterial cells in natural populations [26,47]. Moreover, some bias can be introduced by many mechanical factors, such as sample handling, DNA extraction, qPCR standards preparation and PCR conditions [50].

The present work showed, nevertheless, that qPCR can be used to easily and quickly estimate the toxicity of environmental samples. In fact, toxic genotypes densities correlate well with MC concentrations, suggesting that they could be used to predict MC toxicity risk. This work also showed that as blooms develop, the proportion of toxic genotypes decreases, and that at low densities most of the *Microcystis* genotypes are toxic. These observations indicate that conditions favoring *Microcystis* growth lead to the selection of non-toxic genotypes and, on the other hand, that even at low cell densities there is high potential MC threat. This raises greater concern on the presence of potential MC-producing cyanobacteria in temperate water reservoirs, particularly considering long term exposure effects, making it imperative to develop more efficient monitoring methods.

## 3. Experimental Section

### 3.1. Sample Collection and Analysis of Limnological Parameters

Torrão reservoir is located on the Tâmega River, in the north of Portugal, being used for water consumption and recreational purposes. This reservoir presents periodic dominance of toxin-producing cyanobacteria, including *Microcystis aeruginosa* and *Aphanizomenon flos-aquae* [37]. Surface water samples (0–0.3 m depth) were collected from Marco station (Torrão reservoir), where a water treatment plant is located, every two weeks between July 2009 and September 2009 and then monthly until the end of October. The collected samples were immediately transported cooled to the laboratory. For DNA extraction, volumes of 40 to 2000 mL of well mixed water samples were filtered through GFC filters (Whatman, Kent, UK) in triplicate, and the filters stored at −80 °C. For ELISA analysis, 15 mL of water samples were collected and stored at −20 °C. Limnological parameters were measured *in situ* namely, dissolved oxygen (DO), measured with the oxygen meter Oxi 320 (WTW, Weilheim, Germany), temperature, conductivity and pH, measured with the MultiLine P3 pH/LF (WTW, Weilheim, Germany) instrument.

### 3.2. DNA Extraction and qPCR Reactions

DNA was extracted from the biomass retained in the frozen GFC filters, using a commercially available Kit, PureLink Genomic DNA Mini Kit (Invitrogen, Carlsbad, CA), following the manufacturer’s instructions for gram negative bacteria. The extracted DNA was suspended in 100 μL of elution buffer (pH 8.0) (Invitrogen, Carlsbad, CA) and stored at −20 °C. The reproducibility of the extraction method was evaluated by spectrophotometric quantification of the DNA obtained from the filter replicates. The qPCR reactions were performed using Bio-Rad cycler IQ5 (Bio-Rad, Hercules, CA) with 5 μL of DNA from the standard or sample, 0.4 μM of each primer (0.2 μM for MCYA-CD1F/CD1R and MCYB-2959F/3278R primer pairs) and 1X IQ SYBR Green Supermix (Bio-Rad, Hercules, CA), in a final reaction volume of 25 μL. The CYA 359F/781R primer pair [51] was used to amplify a 422-bp fragment of the 16S rRNA gene common to all cyanobacteria. The Micr 184F/431R [52] was used to amplify a 248-bp fragment of the 16S rRNA gene, specifically from *Microcystis* cells (Table 4).

The mcyA CD1F/mcyA CD1R primer pair [22] was designed to amplify a 297-bp fragment of the *mcyA* gene from MC-synthesizing *Microcystis* and *Planktothrix* strains and was previously proved suitable to detect MC-producing cells from different genera, including *Anabaena*, *Microcystis* and *Planktothrix* (Table 1). The mcyB 2959F/mcyB 3278R primer pair [46] was used to amplify a 320-bp fragment of the *mcyB* gene, which is present in MC-producing strains of *Microcystis*. The thermal cycle conditions are summarized in Table 5. The generation of products was monitored after each extension step by measuring the fluorescence intensity of the double-stranded DNA-binding SYBR Green I dye. All the samples were amplified at least in triplicate. Cycle threshold (Ct) was determined by the fit point method using Bio-Rad IQ5 software (ver. 2.0.148.60623) (Bio-Rad, Hercules, CA). The number of cyanobacterial cells was determined from the Ct obtained according to the regression equations of the external standards. To determine the melting temperatures of the amplification products of the standards and samples, fluorescent melting curve analysis was performed after the PCR by gradually increasing the temperature from 55 °C to 95 °C at a rate of 0.5 °C/cycle. Fluorescence intensity data was collected continuously, and converted to melting peaks using Bio-Rad software. The presence or absence of the amplified product was determined by a melting curve analysis and by subjecting the reaction products to 1.5% (w/v) agarose gel electrophoresis. Ethidium bromide stained bands were visualized on a UV transilluminator (Cleaver Scientific, Warwickshire, UK) and digitally recorded using MicroDOC gel documentation system (Cleaver Scientific, Warwickshire, UK).

### 3.3. Standard Curves Development

A cyanobacterial strain of *M. aeruginosa* (M6) (reference strain) was used for the production of a standard curve. This was based on predetermined cell densities and was established by relating the known DNA concentrations (in cell equivalents) to the Ct values of the diluted samples. 1.5 mL of a suspension containing 2.37 × 10^7^ cells of *M. aeruginosa* (M6) suspension (determined by a direct microscopic count) were collected by centrifugation, and seven dilutions of template DNA ranging from 1:10 to 1:10^6^ (equivalent to 1.2 × 10^6^ cells and 1.2 cells, respectively) were prepared from the DNA extract to serve as external standards of the real-time PCR.

### 3.4. Quantification of MCs by ELISA

The Envirogard^®^ MC Plate Kit (Strategic Diagnostic Inc., Newark, NJ) was used to quantify MC in the water samples collected. This assay uses antibodies against MC-LR, the most common MC, binding also with other MC variants such as MC-RR, MC-YR, or with NOD [53]. Fifteen milliliters of whole water samples were frozen and thawed, and then sonicated on ice for 5 min at 50 W to facilitate cell lysis. The resulting solutions were then applied to the above mentioned ELISA kit following the manufacturer’s instructions. The absorbance was measured using a Synergy™ HT Multi-Detection Microplate Reader (BioTek^®^ Instruments Inc., Winooski, VT) using BioTek’s Gen5™ Data Analysis Software. Values are presented in μg MC-LR equivalents/mL. Previous work has shown that ELISA is efficient for the quantitation of total MC in this reservoir, taken into account the MC chemical profile [54].

### 3.5. Statistical Analysis

The nonparametric Spearman Rank Correlation coefficient was calculated as a measure of correlation between all possible pairs of variables. Statistical analysis was performed using the GraphPad Prism version 5.01 for Windows (GraphPad Software, San Diego, CA) statistical analysis software. Correlations showing *P* value <0.05 were considered significant in this analysis.

## 4. Conclusions

A negative significant correlation was observed between toxic (with *mcy* genes) and non-toxic (without *mcy* genes) genotypes ratio and the overall *Microcystis* density. The results suggest positive selection of non-toxic genotypes under favorable environmental growth conditions. Significant positive correlations could be found between quantity of toxic genotypes and MC concentration, suggesting that the method applied could be useful to predict potential MC toxicity risk. No significant correlation was found between the limnological parameters measured and MC concentrations or toxic genotypes proportions indicating that other abiotic and biotic factors should be governing MC production and toxic genotypes dynamics. The qPCR method here applied is useful to rapidly estimate the potential toxicity of environmental samples and therefore, it may contribute to the more efficient management of water use in eutrophic systems.

## Figures and Tables

**Table 1:** Limnological data of Torrão reservoir during the study period.

Sampling date	01.07.09	15.07.09	30.07.09	12.08.09	26.08.09	01.10.09	28.10.09
**pH**	8.8	8.4	8.1	8.7	7.8	6.1	6.9
**Dissolved oxygen (mg/L)**	-	6.5	10.5	-	4.2	7.0	4.0
**Temperature (°C)**	26.1	25.1	25.2	27.0	25.3	22.8	20.5
**Conductivity (μS/Cm)**	60	63	65	67	74	118	155

**Table 2:** Efficiencies and standard curve parameters obtained by real-time qPCR analysis for the cyanobacteria 16S rRNA, *Microcystis* 16S rRNA, *mcy*B and *mcy*A specific primer sets.

Target gene	Reference strain	Efficiency	Slope	*Y*-intercept	*R^2^*	Melting temperature
**Cyanobacteria 16S rRNA**	*M. aeruginosa* genomic DNA	90.7	−3.568	37.288	0.991	86.5–87.0 °C
***Microcystis*16S rRNA**	*M. aeruginosa* genomic DNA	88.4	−3.636	34.156	0.999	87.5–88.0 °C
**Cyanobacterial*mcy*A**	*M. aeruginosa* genomic DNA	87.6	−3.660	35.092	0.998	82.0–82.5 °C
***Microcystis mcy*B**	*M. aeruginosa* genomic DNA	88.3	−3.637	34.862	1.000	81.5–82.0 °C

**Table 3:** Spearman correlations (*r*) between limnological values and cyanobacteria estimation by qPCR (in bold significant correlations *p* < 0.005).

Spearman correlation values	pH	Dissolved oxygen (mg/L)	Temp. (°C)	Cond. (μS/Cm)	MC-LR (μg eq/L)	Cyanob. 16S rRNA	*Microcystis* 16S rRNA	Cyanob. *mcyA*	*Microcystis mcyB*
**pH**									
**Dissolved oxygen (mg/L)**	−0.31								
**Temperature (°C)**	**0.79**	−0.26							
**Conductivity (μS/Cm)**	−**0.86**	0.14	−0.57						
**Microcystins μg/L**	−0.43	0.20	−0.11	0.71					
**Cyanobacterial 16S rRNA (cell number equivalent/mL)**	−0.29	0.49	0.29	0.50	0.75				
***Microcystis*16S rRNA (cell number equivalent/mL)**	−0.18	0.60	0.36	0.32	0.68	**0.96**			
**Cyanobacterial*mcyA*(cell number equivalent/mL)**	−0.21	0.60	0.21	0.32	**0.79**	**0.86**	**0.93**		
***Microcystis mcyB*(cell number equivalent/mL)**	−0.21	0.60	0.21	0.32	**0.79**	**0.86**	**0.93**	**1.00**	

**Table 4:** Primers used for qPCR quantifications.

Target	Primer	Sequence (5′–3′)	Size (bp)	Reference
**Cyanobacteria 16S rRNA**	Cya 359F Cya 781R	GGGGAATYTTCCGCAATGGG GACTACWGGGGTATCTAATCCCWTT	446	[16]
***Microcystis*16S rRNA**	Micr 184F Micr 431R	GCCGCRAGGTGAAAMCTAA AATCCAAARACCTTCCTCCC	220	[16]
***mcyA*All genera**	*mcyA* CD1F *mcyA* CD1R	AAAATTAAAAGCCGTATCAAA AAAAGTGTTTTATTAGCGGCTCAT	297	[22]
***mcyB Microcystis***	*mcyB* 2959F *mcyB* 3278R	TGGGAAGATGTTCTTCAGGTATCCAA AGAGTGGAAACAATATGATAAGCTAC	350	[46]

**Table 5:** PCR conditions applied with the primers used qPCR quantifications.

PCR protocol	Primer pair				

	Initial denaturation	Denaturation	Annealing	Extension	Number of cycles
CYA 359F/781R	5 min at 95 °C	15 s at 95 °C	15 s at 60 °C	30 s at 72 °C	50
Micr 184F/381R	5 min at 95 °C	15 s at 95 °C	30 s at 52 °C	30 s at 72 °C	40
*mcyA* CD1F/CD1R	5 min at 95 °C	15 s at 95 °C	30 s at 59 °C	30 s at 72 °C	40
*mcyB* 2959F/3278R	5 min at 95 °C	15 s at 95 °C	30 s at 59 °C	30 s at 72 °C	40
